# Enhancing Flexible Neural Probe Performance via Platinum Deposition: Impedance Stability under Various Conditions and In Vivo Neural Signal Monitoring

**DOI:** 10.3390/mi15081058

**Published:** 2024-08-22

**Authors:** Daerl Park, Hyeonyeong Jeong, Jungsik Choi, Juyeon Han, Honglin Piao, Jaehyun Kim, Seonghoon Park, Mingu Song, Dowoo Kim, Jaesuk Sung, Eunji Cheong, Heonjin Choi

**Affiliations:** 1 Department of Materials Science and Engineering, Yonsei University, Seoul 03722, Republic of Korea; dchp513@yonsei.ac.kr (D.P.); wjdtlrsla1@gmail.com (J.C.); han_juyeon@yonsei.ac.kr (J.H.); hongrim@yonsei.ac.kr (H.P.); kimjae5020@yonsei.ac.kr (J.K.); 13chemy@yonsei.ac.kr (S.P.); m5465@yonsei.ac.kr (M.S.); 3720kdw@yonsei.ac.kr (D.K.); 2 Department of Biotechnology, Yonsei University, Seoul 03722, Republic of Korea; griefof@naver.com; 3 Nformare Inc., Seodamun-gu, Seoul 03722, Republic of Korea; sungjs11@naver.com

**Keywords:** flexible printed circuit board (FPCB), low impedance, platinum coating, monitoring, awake animals

## Abstract

Monitoring neural activity in the central nervous system often utilizes silicon-based microelectromechanical system (MEMS) probes. Despite their effectiveness in monitoring, these probes have a fragility issue, limiting their application across various fields. This study introduces flexible printed circuit board (FPCB) neural probes characterized by robust mechanical and electrical properties. The probes demonstrate low impedance after platinum coating, making them suitable for multiunit recordings in awake animals. This capability allows for the simultaneous monitoring of a large population of neurons in the brain, including cluster data. Additionally, these probes exhibit no fractures, mechanical failures, or electrical issues during repeated-bending tests, both during handling and monitoring. Despite the possibility of using this neural probe for signal measurement in awake animals, simply applying a platinum coating may encounter difficulties in chronic tests and other applications. Furthermore, this suggests that FPCB probes can be advanced by any method and serve as an appropriate type of tailorable neural probes for monitoring neural systems in awake animals.

## 1. Introduction

Recently, considerable research has been conducted on the monitoring of neural signals in the brain. However, due to the brittle nature of silicon materials [1,2,3], mechanical mismatches [4], and geometric issues [5], they pose challenges for handling and use in active animals to allow for chronic implantation [6]. As a result, flexible neural probes made of polymers such as polyimide [7,8] or Parylene [9,10], which have excellent biocompatibility and mechanical properties related to flexibility, have been developed.

Most flexible neural probes are microfabricated flexible probes. However, electrodes fabricated using this method often face challenges such as complex manufacturing processes [11] and limitations in electrode configuration [12]. Therefore, there is significant research interest in flexible neural probes utilizing flexible printed circuit boards (FPCBs) to overcome these limitations. FPCBs enable easier fabrication [13] and can be manufactured in various shapes [14], including front–back electrodes and electrodes with both stimulation and recording [15], addressing the challenges.

Although FPCBs have excellent mechanical properties [16], their electrode sites have a high impedance that may not be sufficient for neural recording. To overcome this issue, neural probes are enhanced for recording by reducing impedance through complex processes that require more than 10 steps [17]. Alternatively, materials such as iridium [18] and Zinc compounds [19], known for their high capacitance, are coated to control the impedance. Furthermore, in terms of biocompatibility, conducting polymers [20] such as PEDOT/PSS [21] and polypeptide [22] are employed to coat the electrode, exhibiting excellent impedance characteristics.

Not only these materials but also platinum is coated by many different methods such as electrodeposition [23] or physical vapor deposition [8]. However, these fabricated neural probes need complicated processes or extra treatment, such as annealing or etching. Specifically, when using platinum black coated via electrodeposition, many studies either require additional materials such as iridium [24] or conducting polymer [25]. Additionally, while it is better to conduct both in vitro and in vivo experiments, researchers may inevitably choose to emphasize one over the other based on the specific focus of their study [26,27].

Moreover, various neural probes fabricated using the methods mentioned above exhibit low stability at high temperatures [28,29] and under acidic/basic conditions [30], owing to the properties of the coated materials and the polymer used to fabricate the FPCB.

Here, we used the electrodeposition technique to coat the electrodes of the FPCB with a platinum solution. By coating platinum nanoparticles on the surface of the electrodes, the impedance of the multi-channel FPCB electrodes decreased. We also conducted in vivo experiments to verify the recording capability. We were able to measure signals in awake mice and found an advantage in distinguishing clusters, not just spikes.

Although the platinum-coated FPCB showed good performance in distinguishing clusters, it faced issues with the low success rate of recording unit cells during whisker stimulation tests and only 14 days of tests for chronic usage. To address this problem, additional steps for electrode modification, such as chemical vapor deposition or electrodeposition, are needed. Therefore, we plan to conduct further tests to measure impedance data under various conditions, including high temperatures and acidic/basic environments, to identify any impedance differences.

As a result, we observed changes in the impedance of FPCBs through mechanical-bending tests, thermal tests, and chemical tests under alkaline/acidic conditions. We identified the conditions under which impedance was maintained.

## 2. Materials and Methods

### 2.1. Platinum Deposition of the Neural Probe

**Figure 1:**
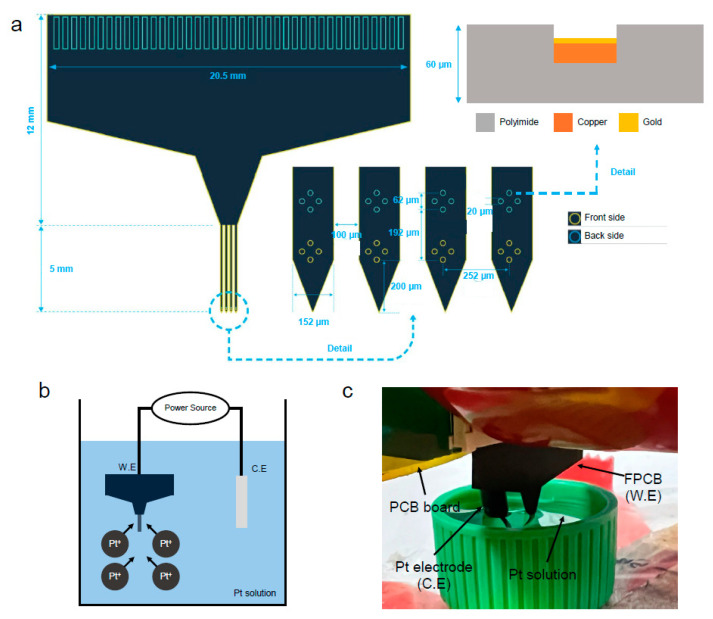
(**a**) Schematic image of N32-1-b probe. (**b**) Schematic image of electro-deposition method of platinum. (**c**) Real image of electro-deposition method of platinum.

A commercial multi-channel FPCB (N32-1-b, Nformare, Seoul, Republic of Korea) was used. This probe has four shanks, and each shank on the FPCB comprises four electrodes as a tetrode type with 20 µm diameter (area of 314 µm^2^), which were placed on both sides of the shank. Each of the shanks is 5 mm long, 152 µm wide, and has total thickness of 60 µm. Most of the FPCB is composed of polyimide (PI), line of the FPCB is composed of 16 µm of copper (Cu), and the electrode is coated by 4 µm of gold (Au). This information and other details of the ‘N32-1-b’ probe are included in Figure 1a.

To decrease the impedance of the FPCB, it is dipped in a platinum solution (H_2_Cl_6_Pt solution, Neuralynx, Bozeman, MT, USA) diluted to a volume ratio of 1:100 of distilled water and placed on the working electrode (W.E). By using two-electrodes system with the platinum (Pt) electrode on the counter electrode (C.E) [31], platinum nanoparticles were coated by repeating Galvanostatic mode of electrodeposition method (Figure 1b,c) using VSP-300 (BioLogic, Seyssinet-Pariset, France) device. The coating condition is as follows: current of 150 µA, coating time of 5 s with 5 s interval, and the cycle is repeated 50 times.

### 2.2. SEM (Scanning Electron Microscopy) and EDS (Energy-Dispersive X-ray Spectroscopy) Analysis

SEM (Scanning Electron Microscopy) and EDS (Energy-Dispersive X-ray Spectroscopy) are commonly used analytical techniques in materials science and engineering to investigate the microstructure and chemical composition of materials. In this study, the SEM and EDS analyses (accelerate voltage: 15 kV) were performed on the fabricated FPCB electrodes to examine the surface morphology and elemental composition of the electrode surface.

### 2.3. Electrochemical Impedance Spectroscopy (EIS) Measurement

To measure the impedance of the multi-channel FPCB, EIS (Electrochemical Impedance Spectroscopy) is used with a VSP-300 (BioLogic) device. The FPCB is connected to the PCB, which can measure the impedance of each channel, and the needle of FPCB is dipped into PBS (Phosphate Buffered Saline) (KR-Thermo Fisher Scientific, Paisley, The Granite City, UK) solution. A box plot with median, maximum, and minimum values is used to compare each sample with all the channels. For reliable measurement of impedance, relative standard deviation (RSD) value was calculated, and ordinary one-way ANOVA test was conducted to compare the impedance. The measuring condition is as follows: the frequency range is 100 Hz to 10,000 Hz. A total of 50 points are measured per decade, 5 measurements are taken per frequency on average, and the sine wave amplitude is 10 mV.

### 2.4. Mechanical Test

**Figure 2:**
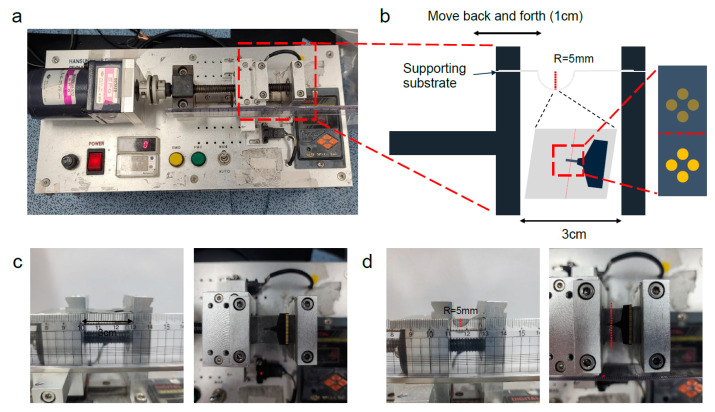
(**a**) Mechanical test device; (**b**) schematic image of mechanical test setting. (**c**) Real image of side view (left) and top view (right) of the initial state of the bending test. (**d**) Real image of side view (left) and top view (right) of the bending state of the bending test.

The mechanical test [32] is performed using a bending test device (Figure 2a). The fabricated FPCB is fixed on the supporting substrate (3M 5069, double-sided acryl foam tape), which has a 1.1 mm thickness. Part of electrode is placed in the middle of the bending position. The substrate is then moved 1 cm, and the electrode of FPCB is bent to around 5 mm bending radius (Figure 2b). The bending distance is determined based on the previous research of reliability of bending test [33,34]. After bending, the substrate is stretched back to initial state. A real image of the bending test is shown in Figure 2c,d. This bending process is repeated 250 and 500 times [35,36]. After the mechanical test, the impedance of each FPCB is compared to sample with 0 bending repetitions.

### 2.5. Thermal Test

The thermal test is performed using a tube furnace. The FPCB is loaded into the tube using a quartz plate. Once loaded, it is heated to 150 °C, which is the lowest temperature that could influence the gold’s electrochemical properties [37], and to 175 °C with a heat rate of 5 °C/minute under 500 sccm Argon conditions. The heat treatment lasts for one hour. After the heat treatment, the FPCB is cooled down to room temperature, and its impedance is compared to a reference sample that is not subjected to heat.

### 2.6. Chemical Test

The chemical test involves the use of acidic/alkaline solution. The alkaline test is conducted using 0.1 M of NaOH solution (Sigma Aldrich, St. Louis, MO, USA), where the electrode of the FPCB is fully immersed (2 mm) in the solution for an hour. After this treatment, the impedance is compared to a neutral reference sample. The acidic test is also conducted by fully dipping the electrode of the FPCB in a 0.1 M HCl solution (Sigma Aldrich, St. Louis, MO, USA) for an hour and comparing the impedance to a neutral reference sample.

### 2.7. In Vivo Electrophysiological Recording

Adult female C57BL/6J mice (12–16-week-old) were used. All mice were maintained under a 12:12 h light–dark cycle (lights on 7:00 A.M.) and had ad libitum access to food and water. Care and handling of animals were in accordance with the guidelines of the Institutional Animal Care and Use Committee at Yonsei University (Seoul, Republic of Korea).

The mice were anesthetized using intraperitoneal injection of urethane (1500 mpk). After the heads were fixed on a stereotaxic device, their scalps were incised, and cranial windows, centered at anterio-posterior (A/P) axis of −1.8 mm and medio-lateral (M/L) −1.8 mm (~2 mm × 2 mm), were created. The dura was carefully removed, and the probe was attached to a micrometric stereotaxic arm and connected to a head stage (HS-32-MUX, Neuralynx, Bozeman, MT, USA), a connector (Nformare, Seoul, Republic of Korea), an adapter (ADPT-HS36-N2T-32, Neuralynx, USA), and a Lablynx recording system (Neuralynx, USA) for data acquisition (Figure S1). The flexible neural probes were lowered through the cranial window using a stereotaxic arm until the probes reached dorso-ventral (D/V) −3.3 mm for Ventral Posterior medial (VPm) thalamus recordings. Stainless wires were installed in the skull above the cerebellum to serve as a reference electrode using screw. Signals from probe were amplified and acquired at 30 kHz. The signals were then filtered between 600 Hz and 6000 Hz. Following acquisition, spike sorting was performed offline using MClust. Additionally, to confirm that neural signal measurement is possible in awake mice, we conducted the same in vivo electrophysiological recording on a mouse after 2 weeks [38].

### 2.8. Whisker Stimulation

The whiskers of mice were glued to the servomotor working arm at a distance of 10 mm from the whisker pad. The working arm protracted the whiskers by 5 degrees for 250 ms and retracted it for 250 ms. Inter-trial intervals were randomly applied between 1 and 6 s. The motor was controlled by the Arduino. The VPm firings upon the working arm movement were measured before and after whisker cutting. To plot the Peri-Stimulus Time Histogram (PSTH), the measured neural responses were binned to 1 ms and smoothed through a Gaussian filter.

## 3. Results

### 3.1. Properties of Neural Probe

The exceptional adhesion and inherent stability of platinum play pivotal roles in sustaining low impedance, even after undergoing repeated-bending tests (mechanical tests), high-temperature tests (thermal tests), and acidic/alkaline tests (chemical tests). Consequently, the neural probe produced through this method, termed the ‘N32-1-b’ probe, enables the accurate recording of real action potentials from mice.

**Figure 3:**
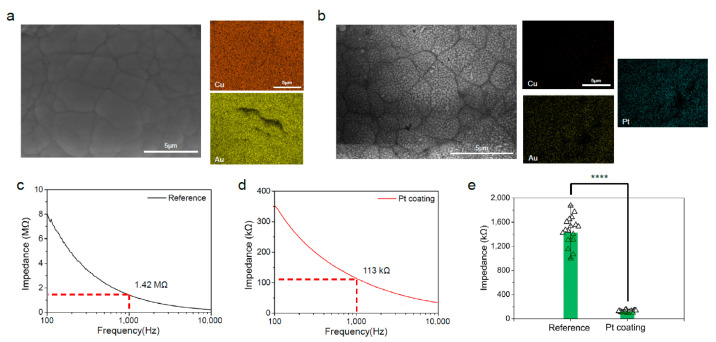
(**a**) SEM image of the electrode of N32-1-b probe (left). EDS mapping data for the same image (right). Orange for copper, and yellow for gold. (**b**) SEM image of the electrode of N32-1-b probe (left) after electrodeposition of platinum. EDS mapping data for the same image (right). Orange for copper, yellow for gold, and light blue for platinum. (**c**) Average impedance data of 32 channels from reference N32-1-b electrode (**d**) Average impedance data of 32 channels from platinum-coated N32-1-b electrode (**e**) Impedance distribution at 1 kHZ of reference N32-1-b electrode (left) and impedance distribution at 1 kHZ of platinum-coated N32-1-b electrode (right). ‘****’ indicates that the observed differences between the groups are highly statistically significant, with a *p*-value of less than 0.0001.

The electrode composition undergoes changes post-electro-deposition. There was a significant difference in morphology due to the deposition of the platinum nano particles, with a diameter between 150 nm and 250 nm (Figure S2). Initially composed of gold and copper, the FPCB’s electrode composition is 74% copper and 26% gold. However, it transforms to 3.91% copper, 20.50% gold, and 75.6% platinum after the platinum solution electro-deposition, which is shown by EDS mapping (Figure 3a,b). Not only does the composition change, but the impedance of the electrode is also altered post-electro-deposition. Due to the increased platinum content, which exhibits a lower impedance than copper [39], the average impedance of the N32-1-b probe experiences a shift.

Prior to coating, the impedance of the FPCB’s electrode at 1 kHz ranged from 998 kΩ to 1.87 MΩ, with an average impedance of approximately 1.42 MΩ (RSD: 17.48%, *n* = 19) (Figure 3c). After platinum coating, the impedance decreased to about 113 kΩ (RSD: 15.52%, *n* = 19), ranging from 95 kΩ to 152 kΩ (Figure 3d). The difference in impedance between the electrodes was significant, with a *p* value of 0.0376 (Figure 3e).

**Figure 4:**
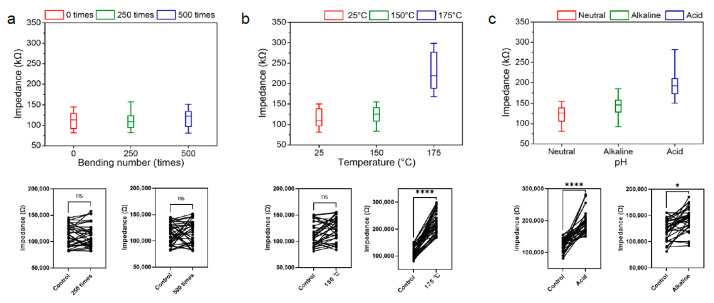
(**a**) Box plot of impedance data before mechanical test (0 times for red) and after mechanical test (250 times for green, 500 times for blue). The sample-by-sample variability and ANOVA test results are shown at the bottom. (**b**) Box plot of impedance data before thermal test (room temperature for red) and after thermal test (150 °C for green, 175 °C for blue). The sample-by-sample variability and ANOVA test result are shown at the bottom. (**c**) Box plot of impedance data before chemical test (neutral for red) and after chemical test (alkaline for green, acidic for blue). The sample-by-sample variability and ANOVA test result are shown at the bottom. ‘ns’ indicates that the observed differences between the groups are not statistically significant, with a *p*-value over than 0.05. ‘*’ indicates that the observed differences between the groups are statistically significant, with a *p*-value of less than 0.05. ‘****’ indicates that the observed differences between the groups are highly statistically significant, with a *p*-value of less than 0.0001.

After the modification, a mechanical test [32] was conducted using a bending test device (Figure 2). Following 250 and 500 bending cycles, the FPCB displayed no apparent changes except for a slight warping of the tip. However, the tip reverted to its initial state when bent in the opposite direction. Moreover, there was no significant change in impedance. After 250 bends, the average impedance of the 32 channels was 112 kΩ (RSD: 19.02%, *n* = 32), and after 500 bends, the average impedance was 117 kΩ (RSD: 19.38%, *n* = 32) (Figure 4a). The difference between the non-bent sample (112 kΩ, RSD: 17.95%, *n* = 32) and the bent sample was compared using an ANOVA test. The difference was within the margin of measurement error, with *p* = 0.998 (*n* = 32) for 250 times and *p* = 0.606 (*n* = 32) for 500 times (Figure 4a). Not only did the average impedance remain constant, but the impedance range also showed no alteration. The non-bent sample had an impedance range of 80 kΩ to 150 kΩ, and the samples subjected to 250 and 500 bends exhibited the same impedance range.

The thermal test was conducted using a tube furnace under an Argon gas atmosphere at 150 °C and 175 °C. Following the heat treatment, the FPCB showed no apparent physical change. Unlike most other FPCBs that may bend due to their different coefficients of thermal expansion [40,41], the N32-1-b probe remained unchanged. In addition, the average impedance of N32-1-b remained unchanged (*p* = 0.476, *n* = 32) at 150 °C annealing (Figure 4b). Comparing these results, the impedance of the reference probe, which is about 115 kΩ (RSD: 19.45%, *n* = 32), changes to 124 kΩ (RSD: 17.41%, *n* = 32) and 228 kΩ (RSD: 19.20%, *n* = 32) at 150 °C and 175 °C, respectively. Due to the FPCB’s expansion and contraction during 175 °C heat treatment, the electrode is slightly cracked (Figure S3). This crack leads to an increase in impedance (*p* < 0.0001, *n* = 32) (Figure 4b) [42], but the range of impedance remains under 300 kΩ. However, the impedance of each channel is sufficiently low to record the action potential of the cells.

The chemical tests involve the use of an alkaline or acidic solution. The FPCB treated in an alkaline solution (0.1 M NaOH) shows no change in size and a slight increase in impedance (*p* = 0.0124, *n* = 32) (Figure 4c) to 143 kΩ (RSD: 16.04%, *n* = 32). Similarly, the FPCB treated in an acidic solution (0.1 M HCl) exhibits no changes in size and increase in impedance (*p* < 0.0001, *n* = 32) to 198 kΩ (RSD: 18.25%, *n* = 32) (Figure 4c). There was an increase in impedance when treated in an acidic solution due to the slight dissolution of platinum [30]. Still, the impedance of each channel remains low enough (below 300 kΩ) to record the action potential of cells.

### 3.2. In Vivo Electrophysiological Recordings

**Figure 5:**
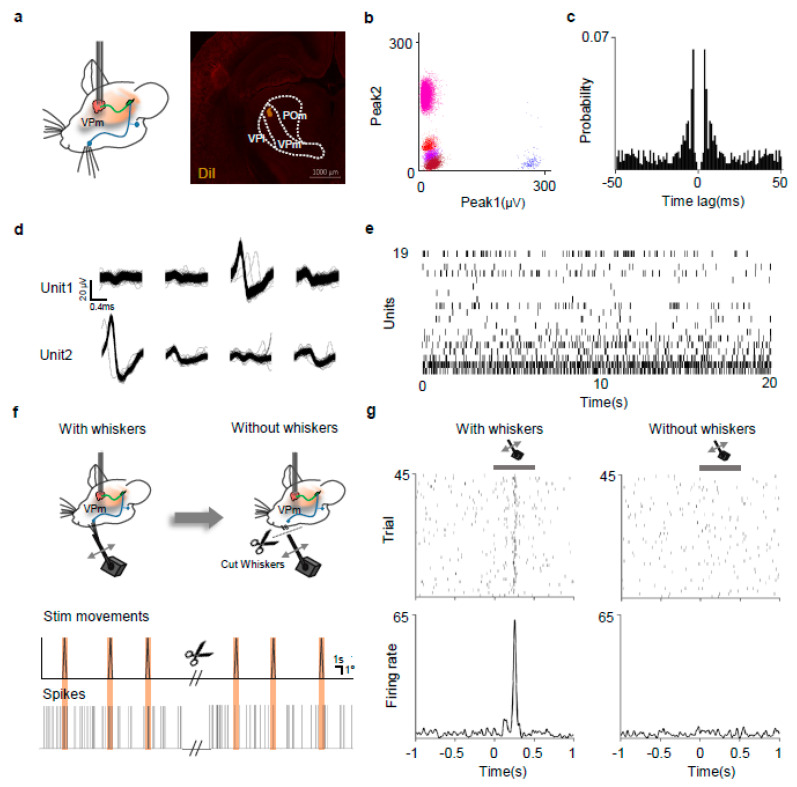
Simultaneous single-unit recording from multiple neurons using the neural probe. (**a**) Schematic of neural recording using the probe (left) and example recording site (right). Orange, DiI Fluorescence in the VPm thalamus. (**b**) Representative clusters of putative action potentials recorded with the probe. (**c**) Autocorrelation of a unit spike times. (**d**) Representative spike waveforms of two units recorded from four adjacent electrode sites. (**e**) Raster plot of neural spikes from total of 19 units. (**f**) Schematic of firing recording in the VPm thalamus upon stimulation before and after whisker cutting (top); example of motor movements (middle) and spikes recorded in VPm (bottom). (**g**) Representative raster plots (top) and PSTH (bottom) of a responsive neuron before (left) and after (right) whisker cutting. Gray line—duration of motor movement. Note that whisker cutting abolished VPm spikes upon stimulation, indicating that the signals are indeed neuronal firings.

To assess the probe’s capability in measuring the firing of single neurons, in vivo electrophysiological recordings were conducted in the Ventral Posterior medial (VPm) thalamus of anesthetized mice, and after the analysis, we histologically confirmed that the probe had been successfully inserted into the VPM (Figure 5a) (N = 2). The recorded signal underwent amplification and was subsequently filtered through a 600–6000 Hz [43] bandpass filter, capturing the putative action potentials of the neurons. To segregate these potentials into distinct units and eliminate noise, signals simultaneously recorded from multiple channels were clustered based on various spike features, leading to noise-removed clusters (Figure 5b,c). The clusters, each consisting of different colors, represent the action potential peak values that are considered to originate from a single unit. The evaluation of these clusters through waveform analysis and autocorrelation (Figure 5d,e) suggested that our probe could effectively monitor the action potentials of distinct neuronal units. Action potentials were successfully recorded from 19 out of 32 electrodes.

The VPm thalamus, a brain region involved in the somatosensory pathway processing whisker stimulus information from the brainstem, was chosen to test the probe’s ability to measure the neural responses to stimuli. Whiskers of anesthetized rats were stimulated using a controlled motor while recording from the VPm thalamus (Figure 5f). Among the recorded neuronal units, 26% (5/19) responded to the whisker stimulus, consistently generating responsive firings trial-by-trial (Figure 5g). To verify that the recorded signals were not artifacts of the motor, the stimulator was operated in the same manner after cutting all whiskers. The trials without whiskers failed to generate responsive firings (Figure 5g). These results affirm that our probe can effectively measure the neuronal responses to stimuli.

**Figure 6:**
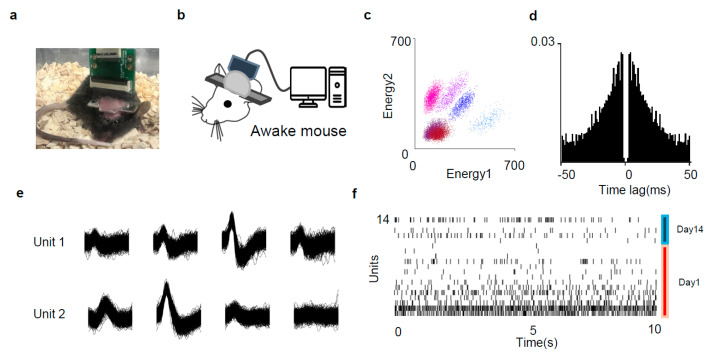
Single-unit recording in awake, head-fixed animal. (**a**) Probe implantation for awake, head-fixed recording. (**b**) Schematic illustration of head-fixed recording. (**c**) Representative activity clusters of 2 electrodes in an awake mouse after implantation. (**d**) Representative autocorrelogram of unit 1. (**e**) Representative spike waveforms of a unit recorded from four adjacent electrode sites at day 1 (top) and day 14 (bottom). (**f**) Representative plot of neural spikes from total of 14 units. The red line is the units 1 day after implantation, and the blue line is the units 14 days after implantation.

To confirm that our probe can measure neural activity in awake animals, we implanted the probe into the mice’s heads (N = 2) with a head post for head fixation (Figure 6a). After implantation, the mice were fixed to the head-fixation system, and neural activity in the VPm thalamus was measured using the probe (Figure 6b). The signals obtained from the electrodes were separated well from each cluster, as well as noise (Figure 6c). Each unit was evaluated to be a single neuron through waveform and autocorrelogram (Figure 6d,e), and separated units showed strong activity (Figure 6f red). In this experiment, nine action potentials were recorded on day 1, and five (55%) were recorded after 14 days. The probe-implanted mice appeared healthy even after two weeks, and the platinum nanoparticles remained on the electrode (Figure S4). To assess whether neural activity could be measured over an extended period, a mouse was fixed to a head-fixation system, and neural activity was measured. Even after two weeks, strong neural activity was observed (Figure 6f blue).

## 4. Discussion

After platinum electrodeposition, platinum nanoparticles are coated onto the electrode (Figure 3a,b). This morphology, characterized by a large effective electrochemical surface area, leads to a decrease in impedance due to reduced resistance and increased capacitance. Consequently, the average impedance at 1 kHz decreased to 113 kΩ (RSD: 15.52%, *n* = 19), compared to 1.42 MΩ (RSD: 17.48%, *n* = 19) before (Figure 3c,d). The impedance is similar to other Si probes, but when comparing impedance per unit area, N32-1-b has the lowest value (Table 1).

Our probe with platinum nanoparticles exhibits different tendencies in impedance changes under various conditions. They possess great mechanical strength, which minimizes the risk of breakage during handling and penetration. Moreover, they can withstand temperatures up to 150 °C. However, at temperatures above 150 °C or under acidic and basic conditions, there are significant differences in impedance. This will serve as a guideline for predicting how certain conditions may alter impedance during additional modification processes when advancing our electrodes.

Our probe with platinum nanoparticles does not achieve the level of impedance reduction demonstrated by other platinum electrode coatings [44], but it still falls within the impedance range of 50 kΩ to 1 MΩ, which is typical for most neural probes for recording [45]. Furthermore, a lower impedance leads to more accurate recording [46], making our modified neural probe advantageous for recording action potentials. As a result, our neural probe with platinum nanoparticles demonstrates average performance in recording action potentials from multiple cells (19 out of 32).

While the latest probes demonstrate an 83% firing rate [47] and have the capability to measure signals in awake mice for several months [48], our probe is also capable of recording signals from whisker stimulation (Figure 5f,g) and in awake mice for 14 days (Figure 6f) across multiple cells. In an anesthetized state, the probe can be adjusted to the optimal recording position, but in awake mice, the probe is implanted and fixed, making it challenging to adjust to the ideal recording position. This limitation may result in fewer signals (9/32) being captured. Additionally, there was a modest decrease in performance (5/9) (Figure 6f), which is related to platinum detachment, as demonstrated in other studies on platinum electrode coatings [49].

Not only for a few days but also to ensure continuous measurement performance for use as a chronic probe in awake mice, it is essential to minimize damage to the probe itself and overcome performance degradation caused by issues such as immune responses [50] and detachment of the deposited platinum [51]. Although more precise experiments are necessary, simple tests indicate that the coated platinum does not exhibit shedding and elicit additional immune responses (Figures S4 and S5). Moreover, various unknown issues need to be addressed [52], and our ‘N32-1-b probe’ has to overcome these challenges to be suitable for chronic use in awake mice.

**Table 1:** Comparison table between the silicon-based neural probe and our product.

	Neuropixels 2.0	NeuroNexus SiNAPS	N32-1-b
Channel	5120 (1280 per shank)	1024 (128 per shank)	32 (8 for shank)
Size (Electrode)	12 μm × 12 μm (square)	14 μm × 14 μm (square)	20 μm (diameter)
Impedance	~150 kΩ (1.04 kΩ/μm^2^)	~650 kΩ (3.32 kΩ/μm^2^)	~152 kΩ (0.48 kΩ/μm^2^)
Recording rate (Units per electrode)	86% (1100/1280)	25% (260/1024)	59% (19/32)
Durability (Units ratio)	57% (638/1110) for 8 weeks	N/A	55% (5/9) for 2 weeks
Cost	USD 1400	USD 1500	USD 250
Flexibility	Rigid	Rigid	500 times bending stable (R = 5 mm)
Auxiliary device	Head stage, Holder	None	None
Reference	[48,53]	[54]	

## 5. Conclusions

We acknowledge that there are highly advanced Si-based products like Neuropixels [48]. However, still some Si neural probes require a complex protocol [53] and auxiliary devices [55]. They also face challenges in chronic measurements in active animals due to the risk of breakage [56]. In contrast, platinum-coated N32-1-b, which is free of such damage risk, could be more suitable for use in active animals.

Even though our neural probe can record the neural signals for 14 days in awake mice, this duration is still considered short-term for chronic tests (Table 1). As a result, additional modifications to the electrodes are needed, such as employing chemical vapor deposition (CVD) or electrodeposition to create a heterostructure based on the platinum-coated electrode. Most CVD processes involve heat treatment, and electrodeposition methods typically take place in acidic or basic solutions. Therefore, we plan to conduct additional tests to measure impedance data under various conditions to identify any impedance differences.

The platinum-coated N32-1-b is stable enough to withstand heat up to 150 °C, allowing for safe electrode modification through processes such as CVD. Additionally, the platinum-coated N32-1-b is stable under alkaline conditions, making it suitable for modification using methods such as electrodeposition, hydrothermal reactions, and etching in alkaline environments. However, we observed the differences in impedance under conditions of heat treatment at 175 °C and exposure to an alkaline or acidic environment.

We could consider these as milestones for the future advancement of neural probes. This is particularly relevant for contemporary neural probes, which incorporate a platinum-containing heterostructure [57,58] and undergo multiple stages of modification [59]. By recognizing that impedance changes are not solely due to material properties but are influenced by various conditions, we could anticipate and conduct more accurate analyses. This would contribute to the further development of neural probes, including wireless recording systems [60] and experiments for rats [61].

## Data Availability

The data cannot be made publicly available upon publication because no suitable repository exists for hosting data in this field of study. The data that support the findings of this study are available upon reasonable request from the authors.

## Supplementary Materials

The following supporting information can be downloaded at: https://www.mdpi.com/article/10.3390/mi15081058/s1, Figure S1. Real image of mouse recording. Printed circuit board (PCB) are connected to the neural probe. Figure S2. SEM image of various size of Platinum nanoparticles. Figure S3. SEM image of cracks after heat treatment of platinum coated electrode. Figure S4. SEM image of platinum coated electrode after mouse recording. Figure S5. Fluorescence image of the cells around the recording site for day 14. The black area is the path of the neural probe insertion.

## References

[1] Wise K.D., Angell J.B., Starr A. (1970). An Integrated-Circuit Approach to Extracellular Microelectrodes. IEEE Trans. Biomed. Eng..

[2] Wise K.D., Sodagar A.M., Yao Y., Gulari M.N., Perlin G.E., Najafi K. (2008). Microelectrodes, Microelectronics, and Implantable Neural Microsystems. Proc. IEEE.

[3] Fiáth R., Raducanu B.C., Musa S., Andrei A., Lopez C.M., Van Hoof C., Ruther P., Aarts A., Horváth D., Ulbert I. (2018). A Silicon-Based Neural Probe with Densely-Packed Low-Impedance Titanium Nitride Microelectrodes for Ultrahigh-Resolution in Vivo Recordings. Biosens. Bioelectron..

[4] Cointe C., Laborde A., Nowak L.G., Arvanitis D.N., Bourrier D., Bergaud C., Maziz A. (2022). Scalable Batch Fabrication of Ultrathin Flexible Neural Probes Using a Bioresorbable Silk Layer. Microsyst. Nanoeng..

[5] Fiáth R., Márton A.L., Mátyás F., Pinke D., Márton G., Tóth K., Ulbert I. (2019). Slow Insertion of Silicon Probes Improves the Quality of Acute Neuronal Recordings. Sci. Rep..

[6] Chen H., Fang Y. (2023). Recent Developments in Implantable Neural Probe Technologies. MRS Bull..

[7] Takeuchi S., Suzuki T., Mabuchi K., Fujita H. (2003). 3D Flexible Multichannel Neural Probe Array. J. Micromech. Microeng..

[8] van Daal R.J.J., Sun J.-J., Ceyssens F., Michon F., Kraft M., Puers R., Kloosterman F. (2020). System for Recording from Multiple Flexible Polyimide Neural Probes in Freely Behaving Animals. J. Neural Eng..

[9] Takeuchi S., Ziegler D., Yoshida Y., Mabuchi K., Suzuki T. (2005). Parylene Flexible Neural Probes Integrated with Microfluidic Channels. Lab Chip.

[10] Kuo J.T.W., Kim B.J., Hara S.A., Lee C.D., Gutierrez C.A., Hoang T.Q., Meng E. (2013). Novel Flexible Parylene Neural Probe with 3D Sheath Structure for Enhancing Tissue Integration. Lab Chip.

[11] Guo B., Fan Y., Wang M., Cheng Y., Ji B., Chen Y., Wang G. (2021). Flexible Neural Probes with Electrochemical Modified Microelectrodes for Artifact-Free Optogenetic Applications. Int. J. Mol. Sci..

[12] Jeon M., Cho J., Kim Y.K., Jung D., Yoon E.-S., Shin S., Cho I.-J. (2014). Partially Flexible MEMS Neural Probe Composed of Polyimide and Sucrose Gel for Reducing Brain Damage during and after Implantation. J. Micromech. Microeng..

[13] Yang Y., Wu M., Wegener A.J., Vázquez-Guardado A., Efimov A.I., Lie F., Wang T., Ma Y., Banks A., Li Z. (2022). Preparation and Use of Wireless Reprogrammable Multilateral Optogenetic Devices for Behavioral Neuroscience. Nat. Protoc..

[14] Wu Y., Wu M., Vázquez-Guardado A., Kim J., Zhang X., Avila R., Kim J.T., Deng Y., Yu Y., Melzer S. (2022). Wireless Multi-Lateral Optofluidic Microsystems for Real-Time Programmable Optogenetics and Photopharmacology. Nat. Commun..

[15] Kim J.H., Lee G.H., Kim S., Chung H.W., Lee J.H., Lee S.M., Kang C.Y., Lee S.-H. (2018). Flexible Deep Brain Neural Probe for Localized Stimulation and Detection with Metal Guide. Biosens. Bioelectron..

[16] Zhang S., Wang C., Linghu C., Wang S., Song J. (2021). Mechanics Strategies for Implantation of Flexible Neural Probes. J. Appl. Mech..

[17] Pimenta S., Rodrigues J.A., Machado F., Ribeiro J.F., Maciel M.J., Bondarchuk O., Monteiro P., Gaspar J., Correia J.H., Jacinto L. (2021). Double-Layer Flexible Neural Probe with Closely Spaced Electrodes for High-Density in Vivo Brain Recordings. Front. Neurosci..

[18] Fomani A.A., Mansour R.R. (2011). Fabrication and Characterization of the Flexible Neural Microprobes with Improved Structural Design. Sens. Actuators A Phys..

[19] Ryu M., Yang J.H., Ahn Y., Sim M., Lee K.H., Kim K., Lee T., Yoo S.-J., Kim S.Y., Moon C. (2017). Enhancement of Interface Characteristics of Neural Probe Based on Graphene, ZnO Nanowires, and Conducting Polymer PEDOT. ACS Appl. Mater. Interfaces.

[20] Zhang W., Zhou X., He Y., Xu L., Xie J. (2021). Implanting Mechanics of PEG/DEX Coated Flexible Neural Probe: Impacts of Fabricating Methods. Biomed. Microdevices.

[21] Chik G.K.K., Xiao N., Ji X., Tsang A.C.O., Leung G.K.K., Zhang S., Tin C., Chan P.K.L. (2022). Flexible Multichannel Neural Probe Developed by Electropolymerization for Localized Stimulation and Sensing. Adv. Mater. Technol..

[22] Wang M., Fan Y., Li L., Wen F., Guo B., Jin M., Xu J., Zhou Y., Kang X., Ji B. (2022). Flexible Neural Probes with Optical Artifact-Suppressing Modification and Biofriendly Polypeptide Coating. Micromachines.

[23] Freitas J.R., Pimenta S., Santos D.J., Esteves B., Gomes N.M., Correia J.H. (2022). Flexible Neural Probe Fabrication Enhanced with a Low-Temperature Cured Polyimide and Platinum Electrodeposition. Sensors.

[24] Cassar I.R., Yu C., Sambangi J., Lee C.D., Whalen J.J., Petrossians A., Grill W.M. (2019). Electrodeposited Platinum-Iridium Coating Improves in Vivo Recording Performance of Chronically Implanted Microelectrode Arrays. Biomaterials.

[25] Wang L.-C., Wang M.-H., Ge C.-F., Ji B.-W., Guo Z.-J., Wang X.-L., Yang B., Li C.-Y., Liu J.-Q. (2019). The Use of a Double-Layer Platinum Black-Conducting Polymer Coating for Improvement of Neural Recording and Mitigation of Photoelectric Artifact. Biosens. Bioelectron..

[26] Ramesh V., Giera B., Karnes J.J., Stratmann N., Schaufler V., Li Y., Rehbock C., Barcikowski S. (2022). Electrophoretic Deposition of Platinum Nanoparticles Using Ethanol-Water Mixtures Significantly Reduces Neural Electrode Impedance. J. Electrochem. Soc..

[27] Park S., Song Y.J., Boo H., Chung T.D. (2010). Nanoporous Pt Microelectrode for Neural Stimulation and Recording: In Vitro Characterization. J. Phys. Chem. C.

[28] Castagnola V., Bayon C., Descamps E., Bergaud C. (2014). Morphology and Conductivity of PEDOT Layers Produced by Different Electrochemical Routes. Synth. Met..

[29] Zhang K., Ma Z., Deng H., Fu Q. (2022). Improving High-Temperature Energy Storage Performance of PI Dielectric Capacitor Films through Boron Nitride Interlayer. Adv. Compos. Hybrid Mater..

[30] Xing L., Hossain M.A., Tian M., Beauchemin D., Adjemian K.T., Jerkiewicz G. (2014). Platinum Electro-Dissolution in Acidic Media upon Potential Cycling. Electrocatalysis.

[31] Harris A.R., Grayden D.B., John S.E. (2023). Electrochemistry in a Two-or Three-Electrode Configuration to Understand Monopolar or Bipolar Configurations of Platinum Bionic Implants. Micromachines.

[32] Li H.U., Jackson T.N. (2016). Flexibility Testing Strategies and Apparatus for Flexible Electronics. IEEE Trans. Electron Devices.

[33] Saleh R., Barth M., Eberhardt W., Zimmermann A. (2021). Bending Setups for Reliability Investigation of Flexible Electronics. Micromachines.

[34] Lee H., Cho K., Kong H., Lee S., Lim J., Kim S. (2021). Electrical Characteristics of Flexible Amorphous Indium–Tin–Gallium–Zinc Oxide Thin-Film Transistors under Repetitive Mechanical Stress. Jpn. J. Appl. Phys..

[35] Happonen T., Häkkinen J., Fabritius T. (2015). Cyclic Bending Reliability of Silk Screen Printed Silver Traces on Plastic and Paper Substrates. IEEE Trans. Device Mater. Reliab..

[36] Li H., Yang J., Cheng J., He T., Wang B. (2020). Flexible Aqueous Ammonium-Ion Full Cell with High Rate Capability and Long Cycle Life. Nano Energy.

[37] Ye Z., Huang H., Xu F., Lu P., Chen Y., Shen J., Ye G., Gao F., Yan B. (2023). Thermal Annealing Effect on Surface-Enhanced Raman Scattering of Gold Films Deposited on Liquid Substrates. Molecules.

[38] Lu C., Park S., Richner T.J., Derry A., Brown I., Hou C., Rao S., Kang J., Moritz C.T., Fink Y. (2017). Flexible and Stretchable Nanowire-Coated Fibers for Optoelectronic Probing of Spinal Cord Circuits. Sci. Adv..

[39] Fan B., Wolfrum B., Robinson J.T. (2021). Impedance Scaling for Gold and Platinum Microelectrodes. J. Neural Eng..

[40] Nix F.C., MacNair D. (1941). The Thermal Expansion of Pure Metals: Copper, Gold, Aluminum, Nickel, and Iron. Phys. Rev..

[41] Shi S., Yao L., Ma P., Jiao Y., Zheng X., Ning D., Chen M., Sui F., Liu H., Yang C. (2021). Recent Progress in the High-Temperature-Resistant PI Substrate with Low CTE for CIGS Thin-Film Solar Cells. Mater. Today Energy.

[42] Bosch R.-W. (2005). Electrochemical Impedance Spectroscopy for the Detection of Stress Corrosion Cracks in Aqueous Corrosion Systems at Ambient and High Temperature. Corros. Sci..

[43] Quiroga R.Q., Nadasdy Z., Ben-Shaul Y. (2004). Unsupervised Spike Detection and Sorting with Wavelets and Superparamagnetic Clustering. Neural Comput..

[44] Boehler C., Vieira D.M., Egert U., Asplund M. (2020). NanoPt—A Nanostructured Electrode Coating for Neural Recording and Microstimulation. ACS Appl. Mater. Interfaces.

[45] Cogan S.F. (2008). Neural Stimulation and Recording Electrodes. Annu. Rev. Biomed. Eng..

[46] Johnson M.D., Otto K.J., Kipke D.R. (2005). Repeated Voltage Biasing Improves Unit Recordings by Reducing Resistive Tissue Impedances. IEEE Trans. Neural Syst. Rehabil. Eng..

[47] Beckinghausen J., Ortiz-Guzman J., Lin T., Bachman B., Salazar Leon L.E., Liu Y., Heck D.H., Arenkiel B.R., Sillitoe R.V. (2023). The Cerebellum Contributes to Generalized Seizures by Altering Activity in the Ventral Posteromedial Nucleus. Commun. Biol..

[48] Steinmetz N.A., Aydin C., Lebedeva A., Okun M., Pachitariu M., Bauza M., Beau M., Bhagat J., Böhm C., Broux M. (2021). Neuropixels 2.0: A Miniaturized High-Density Probe for Stable, Long-Term Brain Recordings. Science.

[49] Boehler C., Oberueber F., Stieglitz T., Asplund M. (2017). Nanostructured Platinum as an Electrochemically and Mechanically Stable Electrode Coating. Proceedings of the 2017 39th Annual International Conference of the IEEE Engineering in Medicine and Biology Society (EMBC).

[50] Xu J., Wang J., Qiu J., Liu H., Wang Y., Cui Y., Humphry R., Wang N., DurKan C., Chen Y. (2021). Nanoparticles Retard Immune Cells Recruitment in Vivo by Inhibiting Chemokine Expression. Biomaterials.

[51] Ferlauto L., Vagni P., Fanelli A., Zollinger E.G., Monsorno K., Paolicelli R.C., Ghezzi D. (2021). All-Polymeric Transient Neural Probe for Prolonged in-Vivo Electrophysiological Recordings. Biomaterials.

[52] Mols K., Musa S., Nuttin B., Lagae L., Bonin V. (2017). In Vivo Characterization of the Electrophysiological and Astrocytic Responses to a Silicon Neuroprobe Implanted in the Mouse Neocortex. Sci. Rep..

[53] van Daal R.J.J., Aydin Ç., Michon F., Aarts A.A.A., Kraft M., Kloosterman F., Haesler S. (2021). Implantation of Neuropixels Probes for Chronic Recording of Neuronal Activity in Freely Behaving Mice and Rats. Nat. Protoc..

[54] Berdondini L., Angotzi G.N., Voroslakos M., Perentos N., Ribeiro J.F., Vincenzi M., Boi F., Lecomte A., Orban G., Genewsky A. (2024). Multi-Shank 1024 Channels Active SiNAPS Probe for Large Multi-Regional Topographical Electrophysiological Mapping of Neural Dynamics. Res. Sq..

[55] Novais A., Calaza C., Fernandes J., Fonseca H., Monteiro P., Gaspar J., Jacinto L. (2021). Hybrid Multisite Silicon Neural Probe with Integrated Flexible Connector for Interchangeable Packaging. Sensors.

[56] Ulyanova A.V., Cottone C., Adam C.D., Gagnon K.G., Cullen D.K., Holtzman T., Jamieson B.G., Koch P.F., Chen H.I., Johnson V.E. (2019). Multichannel Silicon Probes for Awake Hippocampal Recordings in Large Animals. Front. Neurosci..

[57] Ashraf G., Asif M., Aziz A., Iftikhar T., Liu H. (2021). Rice-Spikelet-like Copper Oxide Decorated with Platinum Stranded in the CNT Network for Electrochemical in Vitro Detection of Serotonin. ACS Appl. Mater. Interfaces.

[58] Zhang Q., Liu Z., Li B., Mu L., Sheng K., Xiong Y., Cheng J., Zhou J., Xiong Z., Zhou L. (2024). Platinum-Loaded Cerium Oxide Capable of Repairing Neuronal Homeostasis for Cerebral Ischemia-Reperfusion Injury Therapy. Adv. Healthc. Mater..

[59] Li J., Liu S., Dong H., Li Y., Liu Q., Wang S., Wang P., Li Y., Li Y., Wei Q. (2023). A ZnIn_2_S_4_/Ag_2_CO_3_ Z-Scheme Heterostructure-Based Photoelectrochemical Biosensor for Neuron-Specific Enolase. Anal. Bioanal. Chem..

[60] Shin H., Byun J., Roh D., Choi N., Shin H.-S., Cho I.-J. (2022). Interference-Free, Lightweight Wireless Neural Probe System for Investigating Brain Activity during Natural Competition. Biosens. Bioelectron..

[61] Angelov S.D., Rehbock C., Ramesh V., Heissler H.E., Alam M., Barcikowski S., Schwabe K., Krauss J.K. (2024). Coating of Neural Electrodes with Platinum Nanoparticles Reduces and Stabilizes Impedance In Vitro and In Vivo in a Rat Model. Coatings.

