# Supplementary material for: Enhancing Flexible Neural Probe Performance via Platinum Deposition: Impedance Stability under Various Conditions and In Vivo Neural Signal Monitoring
Source: Micromachines (Basel). 2024 Aug 22;15(8):1058. doi: 10.3390/mi15081058 (PMC11356038; doi:10.3390/mi15081058)
Supplement: Supplementary file 1 [file micromachines-15-01058-s001.zip › micromachines-3114693-supplementary.pdf]

Supporting Information

# Enhancing Flexible Neural Probe Performance via Platinum Deposition: Impedance Stability under Various Conditions and In-vivo Neural Signal Monitoring

**Daerl Park[1†], Hyeonyeong Jeong[2†], Jungsik Choi[1], Juyeon Han[1], Honglin Piao[1], Jaehyun Kim[1], Seonghoon Park[1], Mingu Song[1], Dowoo Kim[1], Jaesuk Sung[3], Eunji Cheong[2], and Heon-Jin Choi[1,3*]**

[1] *Department of Materials Science and Engineering, Yonsei University,Seoul 03722, Korea*

[2] *Department of Biotechnology, Yonsei University, Seoul 03722, South Korea*

[3] *Nformare Inc., Seodamun-gu, Seoul 120-749, South Korea*

*Corresponding authors

Prof. Heon-Jin Choi: hjc@yonsei.ac.kr

Prof. Eunji Cheong: eunjicheong@yonsei.ac.kr

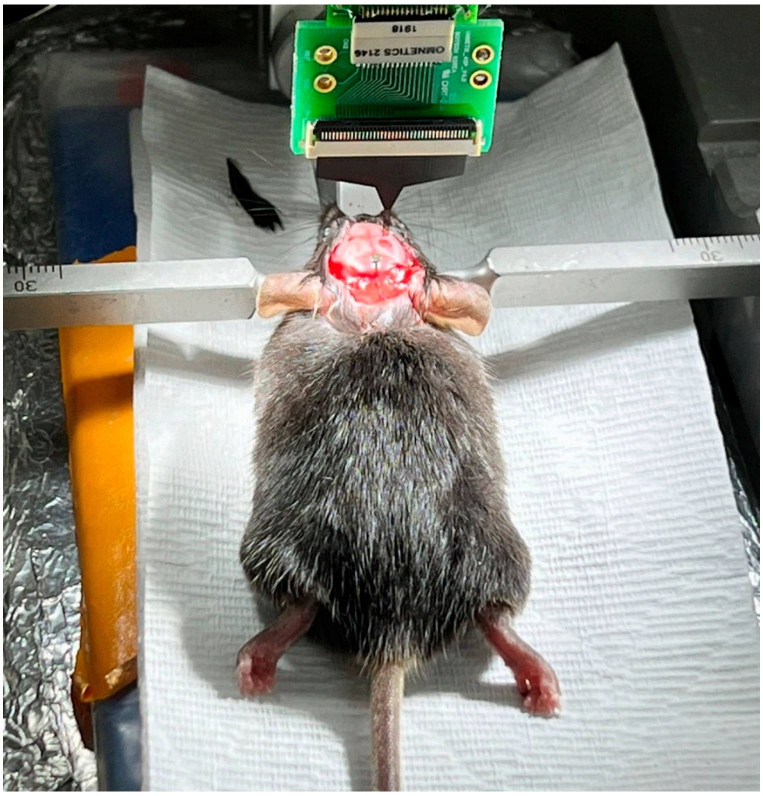


**Figure S1.** Real image of mouse recording. Printed circuit board (PCB) are connected to the neural probe.

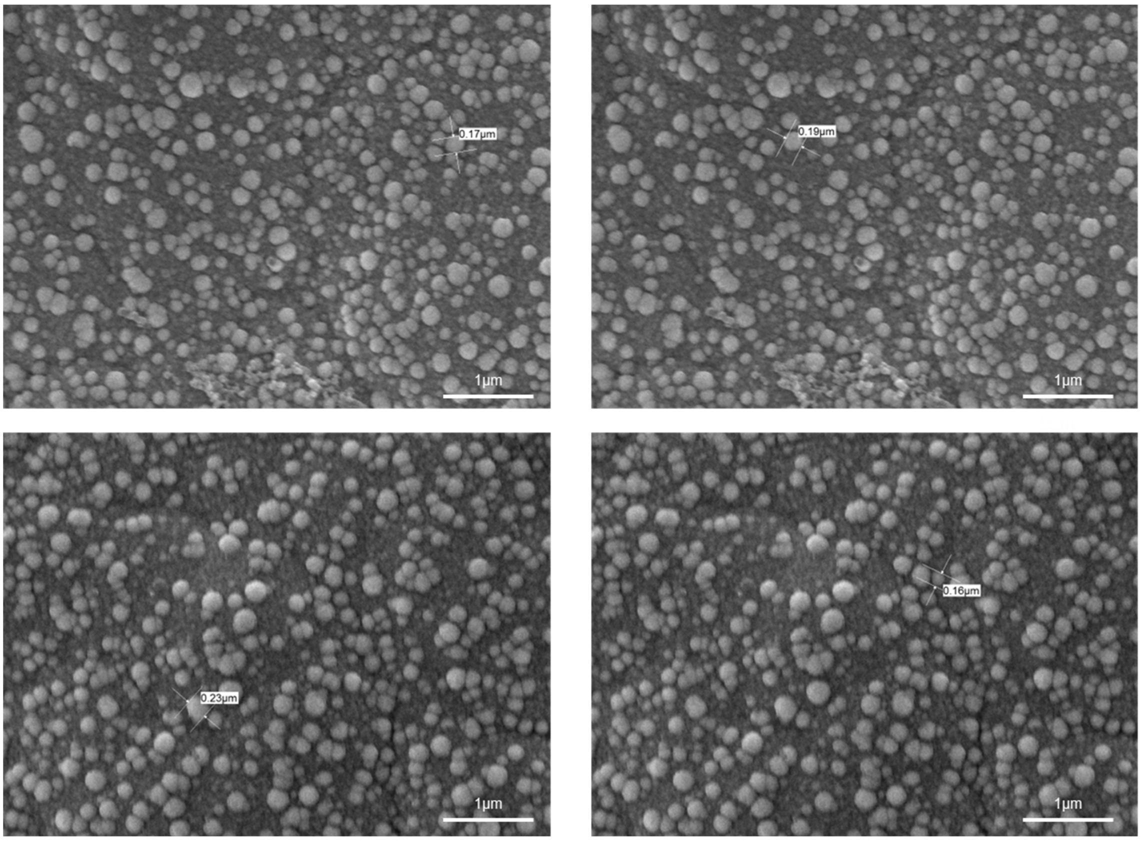


**Figure S2.** SEM image of various size of Platinum nanoparticles.

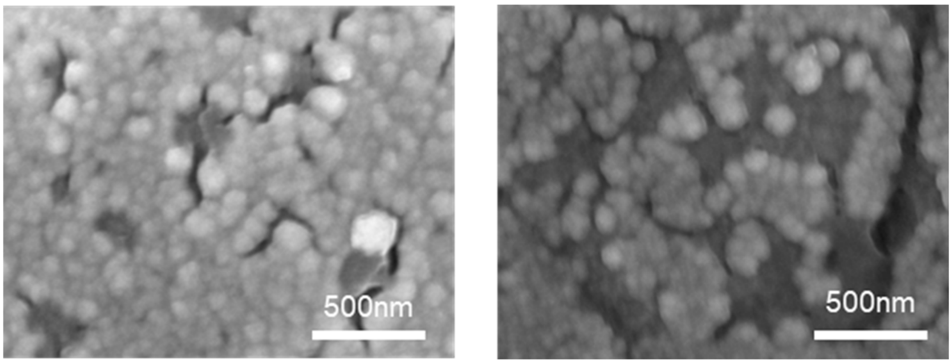


**Figure S3.** SEM image of cracks after heat treatment of platinum coated electrode.

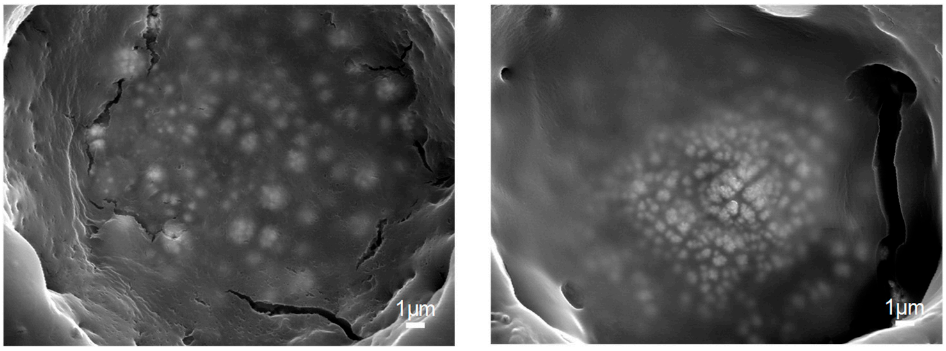


**Figure S4.** SEM image of platinum coated electrode after mouse recording.

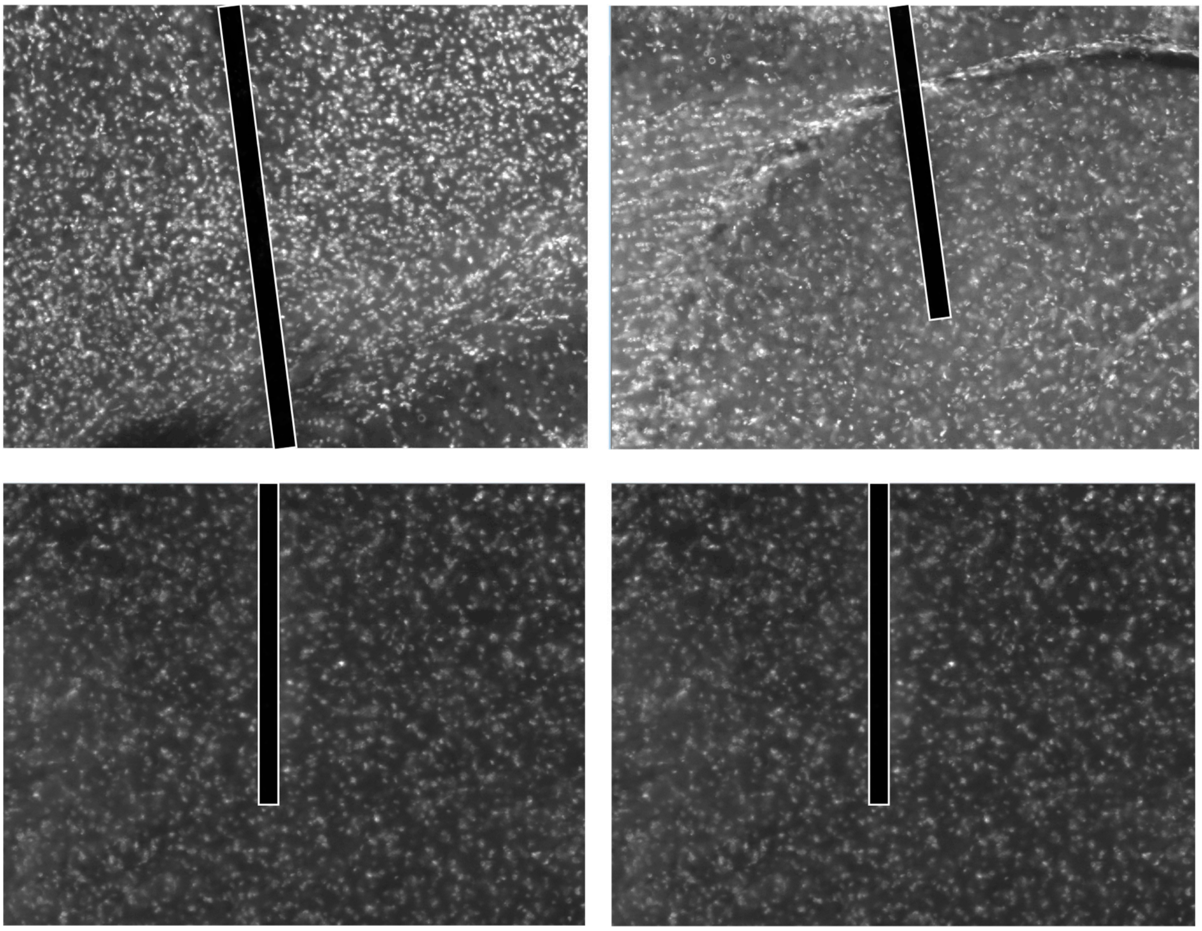

**Figure S5.** Fluorescence image of the cells around the recording site for day 14. The black area is the path of the neural probe insertion.
